# Environmental vinyl chloride exposure and liver comorbidities in hepatocellular carcinoma: a retrospective cross-sectional study

**DOI:** 10.1097/MS9.0000000000005730

**Published:** 2026-10-13

**Authors:** Augustine Ogwoh, Naman Gupta, Heather Hoffman, Aiste Gulla

**Affiliations:** a Department of Biostatistics and Bioinformatics, The George Washington University Milken Institute School of Public Health, Washington, DC, USA; b Department of Surgery, School of Medicine and Health Sciences, George Washington University, Washington, DC, USA

**Keywords:** cross-sectional study, diabetes mellitus, environmental exposure, epidemiology, hepatic fibrosis, hepatocellular carcinoma, United States, vinyl chloride

## Abstract

**Background:**

Vinyl chloride is a Group 1 carcinogen with established links to hepatocellular carcinoma (HCC). Although occupational studies have demonstrated hepatotoxic effects, including fibrosis and metabolic dysfunction, the relationship between environmental exposure levels and liver-related comorbidities among HCC populations remains unclear. Understanding these associations is important for identifying high-risk populations and informing environmental health policies.

**Methods:**

We conducted a retrospective cross-sectional analysis using data from a national multi-center database. The study included 524 HCC cases from the NIH All of Us Research Program (2020–2023) linked with Environmental Protection Agency AirToxScreen 2020 vinyl chloride exposure data. Hepatic fibrosis and diabetes mellitus were identified using SNOMED-CT codes from electronic health records. State-level vinyl chloride exposure metrics were calculated from census tract-level ambient air concentration data. The primary outcomes were prevalence rates of hepatic fibrosis and diabetes mellitus among HCC patients. Secondary outcomes included patterns of comorbidity co-occurrence. Poisson regression analyses, offset by state-level All of Us participant population size, examined associations of hepatic fibrosis and diabetes status with case counts across 12 states. Individual-level multinomial logistic regression evaluated associations between standardized vinyl chloride exposure metrics and comorbidity patterns, adjusting for age, sex, ethnicity, state, and year of diagnosis.

**Results:**

Among 524 HCC cases, 434 (82.8%) had hepatic fibrosis, 288 (55.0%) had diabetes, and 198 (37.8%) had both conditions. Mean age was 69.4 ± 9.3 years; 312 (59.5%) were male, and 403 (76.9%) were non-Hispanic/Latino. The racial distribution included White (257, 49.0%), Black/African American (108, 20.6%), Asian and other races (10, 1.9%), and Other race (149, 28.4%), while 106 participants (20.2%) identified as Hispanic/Latino. State-level vinyl chloride concentrations ranged from 0.000141 to 0.00239 µg/m^3^. Poisson regression demonstrated that individuals with hepatic fibrosis had significantly higher case counts than those without (IRR = 4.96, 95% CI: 3.88–6.42, *P* < 0.001), while diabetes status showed no significant association (IRR = 1.17, 95% CI: 0.97–1.42, *P* = 0.094). Multinomial regression among 436 patients demonstrated no significant associations between vinyl chloride concentration and odds of diabetes only (OR = 0.80, 95% CI: 0.00–3.74 × 10^13^, *P* = 0.99) or both conditions (OR = 0.94, 95% CI: 0.70–1.26, *P* = 0.68) compared to hepatic fibrosis only.

**Conclusions:**

Liver-related comorbidities are highly prevalent among patients with HCC. Poisson regression identified a significant association between hepatic fibrosis status and case counts, while diabetes and vinyl chloride exposure analyses showed no significant associations at current ambient levels. These findings suggest that current environmental vinyl chloride concentrations may not be a significant determinant of liver comorbidities in HCC populations. The high prevalence of comorbidities likely reflects established risk factors and shared pathophysiological pathways rather than low-level environmental vinyl chloride exposure. These results support prioritizing evidence-based risk factor modifications, including antiviral therapy, alcohol cessation, weight management, and metabolic syndrome treatment, as well as comprehensive comorbidity management in HCC care. Future research employing individual-level exposure assessments, longitudinal study designs, and expanded geographic coverage may provide additional insights into potential relationships between environmental exposures and liver health outcomes.

## Introduction

Hepatocellular carcinoma (HCC) is the most common primary liver malignancy and the fourth leading cause of cancer-related mortality worldwide^[^1^]^. HCC typically develops in the context of chronic liver disease, with hepatic fibrosis and diabetes mellitus serving as well-established risk factors that co-occur in affected populations^[^2–4^]^. Understanding the environmental determinants of these comorbidities, such as exposure to vinyl chloride (VC) and other air toxins, is essential for comprehensive cancer care and prevention strategies.

HIGHLIGHTS

- This cross-sectional study examined 524 hepatocellular carcinoma (HCC) patients from the NIH All of Us Research Program (2020–2023) to investigate associations between environmental vinyl chloride exposure and liver comorbidities across 12 U.S. states.
- Hepatic fibrosis was highly prevalent (82.8%), and diabetes mellitus affected more than half (55.0%) of patients with HCC.
- Poisson regression analyses revealed a significant association between hepatic fibrosis status and case counts (IRR = 4.96, 95% CI: 3.88–6.42, *P* < 0.001), while diabetes status showed no significant association (IRR = 1.17, 95% CI: 0.97–1.42, *P* = 0.094). Individual-level multinomial logistic regression showed no significant associations between vinyl chloride exposure and comorbidity patterns (all *P* > 0.05).
- Current ambient environmental vinyl chloride concentrations (ranging from 0.000141 to 0.00239 µg/m^3^) appear to be lower than occupational exposure levels and may not significantly contribute to the liver comorbidity burden in HCC populations.
- These findings support the continued prioritization of modifications of established risk factors – including viral hepatitis treatment, alcohol cessation, and metabolic syndrome management – as well as comprehensive comorbidity surveillance in HCC patient care, rather than the assessment of environmental exposures.

Vinyl chloride (VC), a colorless gas primarily used in the production of polyvinyl chloride plastic, has been classified as a Group 1 carcinogen by the International Agency for Research on Cancer^[^5^]^. Occupational studies have demonstrated associations between VC exposure and hepatic angiosarcoma, with emerging evidence linking exposure to the development of HCC^[^6,7^]^. In addition to carcinogenesis, chronic VC exposure has been associated with hepatotoxic effects, including hepatic fibrosis, portal hypertension, and metabolic dysfunction^[^8,9^]^.

The hepatotoxic mechanism of VC involves the formation of reactive metabolites that induce oxidative stress, hepatocyte damage, and inflammatory responses, leading to fibrosis^[^10^]^. These pathophysiological processes share common pathways with non-alcoholic fatty liver disease and diabetes mellitus, suggesting potential synergistic effects in exposed populations^[^11,12^]^. However, most existing research has focused on occupational exposure at levels higher than those encountered in environmental settings^[^6,7^]^.

Environmental VC exposure primarily occurs through air emissions from industrial facilities, with the U.S. Environmental Protection Agency (EPA)’s AirToxScreen program providing comprehensive data on ambient air concentrations across census tracts^[^13^]^. While environmental exposure levels are lower than occupational exposures, the potential for population-level health impacts remains a concern, given the widespread nature of exposure and the vulnerability of individuals with pre-existing liver disease.

The relationship between environmental VC exposure and liver-related comorbidities, specifically hepatic fibrosis and diabetes mellitus, among populations with HCC has not been systematically investigated. Given VC’s classification as a Group 1 carcinogen and its known hepatotoxic effects, we hypothesized that higher environmental exposure levels would be associated with increased prevalence of hepatic fibrosis and diabetes mellitus among patients with HCC. This study aims to address this knowledge gap by examining associations between comorbidity status and case counts with Poisson regression models, offset by state-level population size, alongside individual-level multinomial logistic regression to evaluate VC exposure metrics and comorbidity patterns, using data from the NIH All of Us Research Program and EPA AirToxScreen 2020.

This cross-sectional study has been reported in line with the STROCSS 2025 guidelines^[^14^]^.

## Methods

### Study design

We conducted a retrospective cross-sectional observational study using electronic health record data from the NIH All of Us Research Program. The study examined data from participants diagnosed with HCC between 1 January 2020 and 31 December 2023, with data extraction and analysis performed between July 2025 and October 2025. This study utilized data from the All of Us Research Program, a national precision medicine initiative that collects health data from diverse populations across the United States^[^15^]^. The All of Us Research Program represents a network of healthcare organizations spanning primary, secondary, and tertiary care settings, including both academic medical centers and community healthcare providers. Data were collected from participants across multiple U.S. states through electronic health record systems integrated with the All of Us database. The data collection period spanned from 1 January 2020 to 31 December 2023 for diagnosis dates, with data extraction occurring in July 2025 based on EPA AirToxScreen 2020 assessments, and analysis conducted from July 2025 to October 2025. No deviations from the initial study design plan occurred during the research period, and the cross-sectional design remained consistent throughout the analysis.

The study included a total of 524 patients with HCC who were classified into three comorbidity groups based on the presence or absence of hepatic fibrosis and diabetes mellitus. The Hepatic Fibrosis Only group consisted of 236 patients with documented hepatic fibrosis without diabetes mellitus; the Diabetes Only group included 90 patients with diabetes mellitus without hepatic fibrosis; and the Both Conditions group comprised 198 patients with both hepatic fibrosis and diabetes mellitus. All participants were assigned state-level environmental VC exposure metrics based on their state of residence, derived from EPA AirToxScreen 2020 data. Participants were included if they had a documented diagnosis of HCC identified by ICD-10 codes C22.0 or C22.9 in electronic health records, with diagnosis dates between 1 January 2020 and 31 December 2023, available geographic information at the state level for exposure assessment, and complete demographic data, including age, sex, and ethnicity. Participants were excluded if they had missing geographic identifiers, suppressed state information due to All of Us privacy protection policies, diagnosis dates prior to 2020 to ensure temporal alignment with EPA AirToxScreen 2020 exposure data, or incomplete demographic or diagnostic information.

Subgroups were defined based on two primary stratification approaches: geographic subgroups and comorbidity-pattern subgroups. Participants were stratified by state of residence for state-level analyses. Poisson regression analyses focused on the 12 states with available data, examining associations between comorbidity status and case counts. Poisson regression models with a population-size offset based on the number of All of Us Research Program participants per state were used for state-level analyses, while multinomial logistic regression models examined individual-level associations while adjusting for potential confounders, including age, sex, ethnicity, state of residence, and year of diagnosis.

As this was a cross-sectional study using retrospective electronic health record data, no active prospective follow-up was conducted. Data represented single-time-point assessments for each participant, with exposure and outcome measurements assessed concurrently based on available records through 31 December 2023. No longitudinal surveillance or post-intervention follow-up was conducted as part of this observational study design.

### Sample size considerations

This study utilized all available HCC cases meeting the inclusion criteria from the All of Us Research Program database during the study period, resulting in a final sample of 524 participants. The sample size was determined by data availability rather than prospective power calculations, as this was a secondary data analysis of existing records. The use of all available qualifying participants maximized the utility of existing data while respecting the All of Us Research Program’s privacy protections.

### Intervention and outcomes

This was an observational cross-sectional study with no interventions administered to participants, and therefore, pre-intervention optimization, intervention procedures, intra-intervention considerations, operator details, intervention settings, quality control measures, and post-intervention care were not applicable. Participants were not exposed to any study-related interventions; rather, the study examined naturally occurring environmental exposures and health outcomes documented in existing electronic health records.

The primary outcomes were Hepatic Fibrosis Prevalence and Diabetes Mellitus Prevalence. Hepatic Fibrosis Prevalence was defined as the proportion of HCC patients with documented hepatic fibrosis, identified using SNOMED-CT diagnostic codes encompassing cirrhosis, fibrosis, and related hepatic conditions from electronic health records. Validation of SNOMED-CT coding for liver disease has been demonstrated in multiple electronic health record studies, with high positive predictive values for cirrhosis and fibrosis diagnoses^[^16,17^]^. This was measured as a binary outcome, either present or absent, determined by the presence of qualifying SNOMED-CT codes in electronic health records from 2020 onward, with cross-sectional assessment in which the most recent diagnosis from 2020-2023 was retained for participants with multiple qualifying diagnoses. Diabetes Mellitus Prevalence was defined as the proportion of HCC patients with documented diabetes mellitus, identified using SNOMED-CT codes for type 1 diabetes, type 2 diabetes, and gestational diabetes from electronic health records, with SNOMED-CT coding for diabetes having demonstrated validity in electronic health record research^[^18^]^. This outcome was similarly measured as a binary outcome, determined by the presence of qualifying SNOMED-CT codes in electronic health records from 2020 onward, assessed cross-sectionally with the most recent diagnosis from 2020 to 2023 retained for participants with multiple qualifying diagnoses.

Secondary outcomes included Comorbidity Co-occurrence Patterns and State-level Comorbidity Prevalence Rates. Comorbidity Co-occurrence Patterns classified participants into three categories based on the presence or absence of both conditions: hepatic fibrosis only without diabetes, diabetes only without hepatic fibrosis, or both hepatic fibrosis and diabetes. State-level Comorbidity Prevalence Rates were calculated as the number of HCC cases with each comorbidity divided by the total number of HCC cases within each state, expressed as percentages (Tables 1 and 2).

**Table 1:** State-level hepatic fibrosis case counts and rates.

State	Hepatic fibrosis status	Mean vinyl chloride	Total cases	Rate	Cases_count
Arizona	With hepatic fibrosis	0.000144	101	0.861	87
Arizona	Without hepatic fibrosis	0.000144	101	0.139	14
California	With hepatic fibrosis	0.000163	43	0.884	38
California	Without hepatic fibrosis	0.000163	43	0.116	5
Florida	With hepatic fibrosis	0.000354	40	0.875	35
Florida	Without hepatic fibrosis	0.000354	40	0.125	5
Georgia	With hepatic fibrosis	0.000247	13	0.769	10
Georgia	Without hepatic fibrosis	0.000247	13	0.231	3
Illinois	With hepatic fibrosis	0.000141	49	0.755	37
Illinois	Without hepatic fibrosis	0.000141	49	0.245	12
Louisiana	With hepatic fibrosis	0.000383	3	1.000	3
Louisiana	Without hepatic fibrosis	0.000383	3	0.000	0
Massachusetts	With hepatic fibrosis	0.00239	53	0.830	44
Massachusetts	Without hepatic fibrosis	0.00239	53	0.170	9
Michigan	With hepatic fibrosis	0.000362	22	0.818	18
Michigan	Without hepatic fibrosis	0.000362	22	0.182	4
New York	With hepatic fibrosis	0.000360	51	0.765	39
New York	Without hepatic fibrosis	0.000360	51	0.235	12
Pennsylvania	With hepatic fibrosis	0.000401	32	0.906	29
Pennsylvania	Without hepatic fibrosis	0.000401	32	0.094	3
Texas	With hepatic fibrosis	0.000268	8	0.875	7
Texas	Without hepatic fibrosis	0.000268	8	0.125	1
Wisconsin	With hepatic fibrosis	0.000343	20	0.750	15
Wisconsin	Without hepatic fibrosis	0.000343	20	0.250	5

Rates represent the proportion of hepatocellular carcinoma (HCC) patients with or without hepatic fibrosis within each state and were calculated as Cases_Count divided by total cases. Mean vinyl chloride concentrations (µg/m^3^) represent state-level averages derived from the U.S. Environmental Protection Agency AirToxScreen 2020 database. Case counts reflect the number of HCC patients with documented hepatic fibrosis identified through SNOMED-CT diagnostic codes in electronic health records from the NIH All of Us Research Program (2020–2023). These state-level counts were used in Poisson regression analyses to evaluate associations between hepatic fibrosis status and case counts across states.

**Table 2:** State-level diabetes case counts and rates.

State	Diabetes status	Mean vinyl chloride	Total cases	Rate	Cases_count
Arizona	With diabetes	0.000144	101	0.505	51
Arizona	Without diabetes	0.000144	101	0.495	50
California	With diabetes	0.000163	43	0.488	21
California	Without diabetes	0.000163	43	0.512	22
Florida	With diabetes	0.000354	40	0.575	23
Florida	Without diabetes	0.000354	40	0.425	17
Georgia	With diabetes	0.000152	13	0.615	8
Georgia	Without diabetes	0.000152	13	0.385	5
Illinois	With diabetes	0.000158	49	0.571	28
Illinois	Without diabetes	0.000158	49	0.429	21
Louisiana	With diabetes	0.000139	3	0.333	1
Louisiana	Without diabetes	0.000139	3	0.667	2
Massachusetts	With diabetes	0.000162	53	0.472	25
Massachusetts	Without diabetes	0.000162	53	0.528	28
Michigan	With diabetes	0.000150	22	0.591	13
Michigan	Without diabetes	0.000150	22	0.409	9
New York	With diabetes	0.000155	51	0.647	33
New York	Without diabetes	0.000155	51	0.353	18
Pennsylvania	With diabetes	0.000148	32	0.469	15
Pennsylvania	Without diabetes	0.000148	32	0.531	17
Texas	With diabetes	0.000147	8	0.375	3
Texas	Without diabetes	0.000147	8	0.625	5
Wisconsin	With diabetes	0.000149	20	0.700	14
Wisconsin	Without diabetes	0.000149	20	0.300	6

Rates represent the proportion of hepatocellular carcinoma (HCC) patients with or without diabetes mellitus within each state and were calculated as Cases_Count divided by Total Cases. Mean vinyl chloride concentrations (µg/m^3^) represent state-level averages derived from the U.S. Environmental Protection Agency (EPA) AirToxScreen 2020 database. Diabetes mellitus status was identified using SNOMED-CT diagnostic codes recorded in electronic health records from the NIH All of Us Research Program between 2020 and 2023. These state-level counts were used in Poisson regression analyses to examine associations between diabetes status and case counts across states.

Environmental VC exposure was assessed using ambient air concentration data measured in µg/m^3^ from the EPA AirToxScreen 2020 database and aggregated to the state level^[^13^]^. State-level metrics included mean VC concentration across census tracts within each state, median VC concentration, maximum VC concentration, and mean cancer risk estimates per million population. AirToxScreen calculated cancer risk estimates by multiplying the average long-term exposure concentration by the inhalation unit risk estimate for VC, expressed as estimated lifetime cancer cases per million population^[^13^]^.

All analyses were performed using the R programming language in the All of Us Jupyter Workbench environment, with specific packages and base R functions for Poisson regression and multinomial logistic regression. Poisson regression was selected for state-level analyses because it is appropriate for modeling count data (case counts per state). By including the number of All of Us Research Program participants per state as an offset term (log-transformed), the model accounts for differences in the size of the enrolled population across states, allowing estimation of incidence rate ratios (IRRs) comparing individuals with versus without each comorbidity. Multinomial logistic regression was selected for individual-level analysis because the outcome variable, comorbidity pattern, had three unordered categories, and this method allows simultaneous estimation of odds ratios for multiple outcome categories relative to a reference group while adjusting for potential confounders.

Statistical tests performed included Poisson regression models estimating IRRs with 95% confidence intervals for state-level count analyses, multinomial logistic regression models estimating odds ratios with 95% confidence intervals, and descriptive statistics. Known or potential confounders controlled in the analysis included age, treated as a continuous variable and standardized in the regression models; sex, treated as a categorical variable comparing males to females; ethnicity, treated as a categorical variable comparing Hispanic/Latino to Other; state of residence, treated as a categorical variable across 12 states; and year of diagnosis, treated as a categorical variable spanning 2020, 2021, 2022, and 2023. These variables were controlled by including them as covariates in the multinomial logistic regression models, although state-level analyses using ecological correlations inherently could not control for individual-level confounders, representing a limitation of that analytical approach.

The primary analysis utilized complete-case analysis, including all participants with non-missing data for exposure and outcome variables, with 524 participants included in descriptive analyses and 436 participants in multinomial regression, after excluding states with missing exposure data. Intention-to-treat principles were not applicable, as this was an observational study without intervention. Subgroup analyses included stratification by comorbidity pattern – hepatic fibrosis only, diabetes only, and both conditions – in multinomial regression. The level of statistical significance was set at a two-tailed α = 0.05. Results are presented as Poisson regression IRRs with 95% confidence intervals and *P*-values, and multinomial logistic regression odds ratios with 95% confidence intervals and *P*-values.

## Results

### Participants

The study included 524 patients diagnosed with HCC between 1 January 2020 and 31 December 2023 from the NIH All of Us Research Program database. All participants meeting the inclusion criteria were retained in the analysis, with no participants excluded due to withdrawal, cross-over, or loss to follow-up, as this was a cross-sectional analysis of existing electronic health record data. Participants were excluded if they had missing geographic identifiers, suppressed state information due to privacy policies, or diagnosis dates prior to 2020, although the exact numbers of excluded participants were not available from the All of Us data extraction process, which prefiltered data based on the researcher-specified criteria.

The study population consisted of 524 HCC patients with a mean age of 69.4 ± 9.3 years (Table 3). The cohort was predominantly male, with 312 patients representing 59.5% of the sample. The racial distribution of the study population was White (257, 49.0%), Black/African American (108, 20.6%), Asian and other races (10, 1.9%), and Other race (149, 28.4%), while 106 participants (20.2%) identified as Hispanic/Latino and 403 (76.9%) as non-Hispanic. Geographic distribution showed that the highest number of cases originated from Arizona, with 101 cases representing 19.3% of the sample, followed by a category of “Other States” representing states with smaller case numbers, with 88 cases at 16.8%. Other states with major representation included Massachusetts, with 53 cases at 10.1%, New York, with 51 cases at 9.7%, Illinois, with 49 cases at 9.4%, and California, with 43 cases at 8.2%. Temporal distribution showed diagnosis years distributed as follows: 2020 with 93 cases at 17.7%, 2021 with 121 cases at 23.1%, 2022 with 196 cases at 37.4%, and 2023 with 114 cases at 21.8%.

**Table 3:** Baseline characteristics of study population.

Characteristic	Total (*n* = 524)	Hepatic fibrosis only (*n* = 236)	Diabetes only (*n* = 90)	Both conditions (*n* = 198)
Demographics				
Age, mean (SD)	69.4 (9.3)	68.1 (9.6)	69.6 (10.6)	70.8 (8.1)
Sex, *n* (%)				
Male	312 (59.5)	138 (58.5)	52 (57.8)	122 (61.6)
Female	201 (38.4)	94 (39.8)	35 (38.9)	72 (36.4)
Race, *n* (%)				
White	257 (49.0)	119 (50.4)	49 (54.4)	192 (97.0)
Black/African American	108 (20.6)	47 (19.9)	22 (24.4)	6 (3.0)
Asian	10 (1.9)	6 (2.5)	0 (0.0)	4 (2.0)
Other Race	149 (28.4)	64 (27.1)	19 (21.1)	66 (33.3)
Ethnicity, *n* (%)				
Hispanic/Latino	106 (20.2)	35 (14.8)	16 (17.8)	55 (27.8)
Non-Hispanic	403 (76.9)	192 (81.4)	73 (81.1)	138 (69.7)
Other/Unknown	15 (2.9)	9 (3.8)	1 (1.1)	5 (2.5)
Geographic distribution – state of residence, *n* (%)				
Arizona	101 (19.3)	50 (21.2)	14 (15.6)	37 (18.7)
California	43 (8.2)	22 (9.3)	5 (5.6)	16 (8.1)
Florida	40 (7.6)	17 (7.2)	5 (5.6)	18 (9.1)
Georgia	13 (2.5)	5 (2.1)	3 (3.3)	5 (2.5)
Illinois	49 (9.4)	21 (8.9)	12 (13.3)	16 (8.1)
Louisiana	3 (0.6)	2 (0.8)	0 (0.0)	1 (0.5)
Massachusetts	53 (10.1)	28 (11.9)	9 (10.0)	16 (8.1)
Michigan	22 (4.2)	9 (3.8)	4 (4.4)	9 (4.5)
New York	51 (9.7)	18 (7.6)	12 (13.3)	21 (10.6)
Pennsylvania	32 (6.1)	17 (7.2)	3 (3.3)	12 (6.1)
Texas	8 (1.5)	5 (2.1)	1 (1.1)	2 (1.0)
Wisconsin	20 (3.8)	6 (2.5)	5 (5.6)	9 (4.5)
Other States	89 (17.0)	36 (15.3)	17 (18.9)	36 (18.2)
Diagnosis year, *n* (%)				
2020	93 (17.7)	48 (20.3)	13 (14.4)	32 (16.2)
2021	121 (23.1)	52 (22.0)	17 (18.9)	52 (26.3)
2022	196 (37.4)	82 (34.7)	41 (45.6)	73 (36.9)
2023	114 (21.8)	54 (22.9)	19 (21.1)	41 (20.7)

This table summarizes the baseline characteristics of the 524 hepatocellular carcinoma (HCC) patients included in the analysis. Patients were stratified by comorbidity status into three groups: hepatic fibrosis only (*n* = 236), diabetes only (*n* = 90), and both hepatic fibrosis and diabetes (*n* = 198). Variables included age at diagnosis (mean ± SD), sex, race, ethnicity, geographic distribution by state of residence, and year of diagnosis (2020–2023). Percentages were calculated within each subgroup unless otherwise indicated.

Baseline characteristics stratified by comorbidity pattern groups showed that the three groups – hepatic fibrosis only, diabetes only, and both conditions – demonstrated comparable demographic characteristics (Table 3). No formal matching procedures were performed because this was an observational study examining naturally occurring comorbidity patterns, and all patients who met the inclusion criteria were assigned to groups based on their documented diagnoses. Principal findings from baseline comparisons revealed that age increased slightly across groups from hepatic fibrosis only to both conditions, though all groups had mean ages within 3 years of each other, while sex and ethnicity distributions were similar across all groups. Geographic and temporal distributions showed some variation but no clear systematic patterns.

### Outcomes

Among the 524 HCC patients, the overall prevalence of liver-related comorbidities was high (Table 3). Hepatic fibrosis was present in 434 patients, representing 82.8% of the cohort; diabetes mellitus affected 288 patients, representing 55.0% of the sample; and both conditions co-occurred in 198 patients, representing 37.8% of the population. State-level variation in comorbidity prevalence was substantial across the 12 geographic areas analyzed (Tables 1 and 2). The high prevalence rate of liver-related comorbidities was consistent with the known associations between chronic liver disease, metabolic dysfunction, and HCC development^[^2–4^]^.

The environmental exposure distribution showed that state-level VC concentrations ranged from 0.000141 µg/m^3^ in Illinois to 0.00239 µg/m^3^ in Massachusetts (Table 4). Massachusetts was identified as a notable outlier, with VC concentrations nearly six times that of the next highest state, Pennsylvania, which measured 0.000401 µg/m^3^. Cancer risk estimates were relatively uniform across most states, ranging from 18.7 to 20.0 cases per million, though Massachusetts showed an anomalous pattern with 10.0 cases per million despite having the highest concentration, suggesting potential differences in population characteristics or risk calculation methodologies.

**Table 4:** State-level vinyl chloride exposure metrics.

State	Mean vinyl chloride (µg/m^3^)	Median vinyl chloride (µg/m^3^)	Maximum vinyl chloride (µg/m^3^)	Mean cancer risk (per million)
Massachusetts	0.00239	0.00225	0.00324	10.0
Pennsylvania	0.000401	0.000461	0.000558	20.0
Louisiana	0.000383	0.000431	0.000555	20.0
Michigan	0.000362	0.000353	0.0539	18.7
New York	0.000360	0.000404	0.0132	19.7
Florida	0.000354	0.000415	0.000558	20.0
Wisconsin	0.000343	0.000363	0.0128	19.5
Texas	0.000268	0.000253	0.000642	19.8
Georgia	0.000247	0.000242	0.000558	20.0
California	0.000163	0.000120	0.00110	19.7
Arizona	0.000144	0.0000814	0.122	17.6
Illinois	0.000141	0.0000653	0.000557	20.0

State-level vinyl chloride exposure estimates were obtained from the U.S. Environmental Protection Agency’s 2020 AirToxScreen database for the 12 states included in the analysis. Metrics included mean, median, and maximum ambient vinyl chloride concentrations (µg/m^3^) and mean estimated lifetime cancer risk per million individuals. These aggregated exposure values were used in the correlation and regression analyses to examine the associations with comorbidity prevalence among patients with HCC.

Poisson regression analyses, offset by the state-level All of Us participant population size, revealed a highly significant association between hepatic fibrosis status and case counts (Table 5). Individuals with hepatic fibrosis had approximately 4.96 times higher case counts than those without hepatic fibrosis (IRR = 4.96, 95% CI: 3.88–6.42, *P* < 0.001). In contrast, Poisson regression for diabetes status showed no significant association with case counts (IRR = 1.17, 95% CI: 0.97–1.42, *P* = 0.094), with the 95% confidence interval crossing 1.0. These analyses were offset by the number of All of Us program participants per state to account for interstate variation in enrolled population sizes, allowing a meaningful comparison of case rates across states.

**Table 5:** Poisson regression analysis results for hepatic fibrosis and diabetes.

Condition	Status	IRR (95% CI)	*P*-value	AIC (with offset)
Hepatic fibrosis	With vs. without fibrosis	4.96 (3.88–6.42)	<0.001***	178.69
Diabetes	With vs. without diabetes	1.17 (0.97–1.42)	0.094 (NS)	333.85

Poisson regression models were used to estimate incidence rate ratios (IRRs) and 95% confidence intervals for associations between comorbidity status and state-level case counts among hepatocellular carcinoma (HCC) patients. Models included a log-transformed offset term representing the number of All of Us Research Program participants per state to account for differences in enrolled population size. IRRs compare case counts among individuals with versus without each comorbidity (hepatic fibrosis or diabetes mellitus). Statistical significance was assessed using a two-tailed α = 0.05. AIC, Akaike Information Criterion; IRR, incidence rate ratio; NS, not significant.

Individual-level multinomial regression analysis conducted among 436 participants examined associations between standardized VC exposure metrics and comorbidity patterns, using “hepatic fibrosis only” as the reference category, with 201 participants representing 46.1% of this subset (Table 6). For the VC concentration model, diabetes only versus hepatic fibrosis only showed an OR of 0.798 with a 95% CI from 0.00 to 3.74 × 10^13^ and *P* = 0.9888, while both conditions versus hepatic fibrosis only demonstrated an OR of 0.941 with a 95% CI from 0.704 to 1.256 and *P* = 0.6784. For the cancer risk estimate model, diabetes only versus hepatic fibrosis only showed an OR of 0.506 with a 95% CI from 0.00 to 5.84 × 10^30^ and *P* = 0.9851, while both conditions versus hepatic fibrosis only demonstrated an OR of 1.072 with a 95% CI from 0.776 to 1.482 and *P* = 0.6719. The extremely wide confidence intervals observed for the “diabetes only” comparison in both models (spanning from near zero to magnitudes of 10^13^ and 10^30^) are indicative of model convergence limitations attributable to the sparse cell size in the diabetes only group (*n* = 90); these estimates should therefore be interpreted with caution. The “both conditions” comparison, which involved a larger cell size (*n* = 198), yielded stable, interpretable confidence intervals. Across both models, all odds ratios were close to the null value of 1.0, indicating no significant associations between VC exposure and comorbidity patterns after controlling for age, sex, ethnicity, state, and year of diagnosis.

**Table 6:** Multinomial logistic regression results: vinyl chloride exposure and comorbidity patterns among HCC patients.

Exposure metric	Outcome	Odds ratio	95% CI	*P*-value
Vinyl chloride concentration (standardized)	Diabetes only vs hepatic fibrosis only	0.798	0–3.74e^+13^	0.9888
	Both conditions vs hepatic fibrosis only	0.941	0.704–1.256	0.6784
Cancer risk (standardized)	Diabetes only vs hepatic fibrosis only	0.506	0–5.84e^+30^	0.9851
	Both conditions vs hepatic fibrosis only	1.072	0.776–1.482	0.6719

Reference category: hepatic fibrosis only (*n* = 201, 46.1%). Total sample: *n* = 436 patients from 12 states. Models adjusted for age, sex, ethnicity, state of residence, and year of diagnosis. Vinyl chloride model AIC: 936.9; Cancer risk model AIC: 936.9. Note: The extremely wide confidence intervals for the diabetes only comparisons reflect model convergence limitations due to sparse cell size (*n* = 90) and should be interpreted with caution.

Confounding assessment through adjustment for demographic and geographic covariates did not reveal any VC-related associations in the multinomial regression models. The Poisson regression analysis confirmed the expected epidemiological association between hepatic fibrosis status and case counts (IRR = 4.96), reflecting the biological link between fibrosis and HCC rather than an environmental VC effect. This significant association is consistent with the well-established pathophysiology in which chronic hepatic fibrosis is a precursor and risk factor for HCC development, rather than a consequence of VC exposure at current ambient levels. All outcomes were assessed cross-sectionally using the most recent diagnoses recorded in electronic health records between 2020 and 2023, although the temporal relationship between exposure and outcome development could not be determined from this study design.

### Tolerance

Assessment of participants’ tolerance or adherence to interventions was not applicable to this observational cross-sectional study, as no interventions were administered. Loss to follow-up was similarly not applicable, as all participants who met the inclusion criteria and had data available at the time of extraction were included in the analysis.

### Complications

No study-related adverse events or complications occurred, as this was a non-interventional observational analysis of existing electronic health records. Adverse event reporting to national agencies or pharmaceutical companies was not applicable.

### Key results

The primary findings revealed a high comorbidity burden among HCC patients, with hepatic fibrosis affecting 82.8% and diabetes mellitus affecting 55.0% of patients, while 37.8% had both conditions simultaneously (Table 3). Poisson regression analyses, offset by state-level All of Us participant population size, revealed a significant association between hepatic fibrosis status and case counts, with an IRR of 4.96 (95% CI: 3.88–6.42, *P* < 0.001) (Table 5). In contrast, diabetes status showed no significant association with case counts (IRR = 1.17, 95% CI: 0.97–1.42, *P* = 0.094).

The Poisson regression findings were robust, with the association for hepatic fibrosis persisting across all modeling approaches. Individual-level multinomial logistic regression (Table 6) demonstrated no significant associations between VC exposure and comorbidity patterns. All odds ratios approximated 1.0, ranging from 0.506 to 1.072, with wide confidence intervals and *P*-values greater than 0.67, indicating no meaningful relationships after adjustment for confounders.

## Discussion

This cross-sectional analysis of 524 HCC patients from the All of Us Research Program showed a high prevalence of liver-related comorbidities. Poisson regression analyses demonstrated a significant association between hepatic fibrosis status and case counts (IRR = 4.96, *P* < 0.001), while diabetes status showed no significant association (IRR = 1.17, *P* = 0.094). Individual-level multinomial logistic regression found no significant associations between VC exposure and comorbidity patterns. Our findings provide important insights into the burden of hepatic fibrosis and diabetes among patients with HCC, while suggesting that current environmental VC exposure may not be a significant determinant of these comorbidities.

The observed prevalence of hepatic fibrosis (82.8%) and diabetes (55.0%) in our HCC cohort aligns with established literature demonstrating the co-occurrence of these conditions in liver cancer populations. The high prevalence of hepatic fibrosis likely reflects the underlying pathophysiology of HCC development, as chronic liver injury and fibrosis are critical steps in hepatocarcinogenesis^[^19^]^. Similarly, the prevalence of diabetes reflects its role as an independent risk factor for HCC and its association with metabolic dysfunction-associated fatty liver disease^[^3,4^]^.

The absence of a significant association between VC exposure and comorbidity patterns may reflect several important biological and methodological considerations. The environmental VC concentrations in our study ranged from 0.000141 to 0.00239 µg/m^3^, which is substantially lower than the occupational exposure levels associated with hepatotoxicity in previous studies^[^6–9^]^. These concentrations are orders of magnitude below occupational exposure standards and may be insufficient to produce measurable population-level health effects, particularly in the context of competing risk factors for liver disease.

Massachusetts was a notable outlier in our analysis, exhibiting the highest VC concentration (0.00239 µg/m^3^) and one of the lowest cancer risk estimates (10 per million). The unique exposure profile of Massachusetts may reflect specific industrial activities, meteorological conditions, or measurement artifacts that differ from those in other states. Despite this outlier profile, Massachusetts did not appear to drive any meaningful exposure-comorbidity associations in either the Poisson or the multinomial regression analyses, with results for hepatic fibrosis and diabetes remaining consistent across all modeled states.

Multinomial logistic regression analysis of 436 patients from 12 states that controlled for age, sex, ethnicity, state of residence, and year of diagnosis provided no evidence of associations between standardized VC exposure metrics and comorbidity patterns (Table 6). The odds ratios clustered around 1.0, indicating no meaningful association between current environmental VC exposure levels and specific comorbidity patterns in patients with HCC.

### Study limitations

The ecological study design inherently limits our ability to assess individual-level exposure-response relationships. State-level aggregation of exposure data may mask important spatial variations in exposure patterns and may fail to account for individual mobility, occupational exposure, or other sources of VC exposure that could influence personal exposure profiles^[^20^]^. Furthermore, the Poisson regression offset was based on the All of Us program’s total population count in the database rather than the true general state population sizes, because such data are not available through the All of Us platform. While this approach appropriately accounts for differences in enrolled population sizes across states, the results should be interpreted as reflecting associations within the All of Us cohort rather than the general population.

It should also be noted that assigning a single state-level mean VC concentration to the participants in the multinomial logistic regression constitutes an ecological fallacy. The same exposure value is attributed to all patients within a given state regardless of their actual residential location, occupational history, or proximity to emission sources. This approach can attenuate or obscure true exposure-response relationships and represents a fundamental limitation of the individual-level regression analysis.

The development of hepatic fibrosis and diabetes in patients with HCC likely involves complex, multifactorial pathways that extend beyond environmental VC exposure. Established risk factors, including viral hepatitis, alcohol consumption, metabolic syndrome, and genetic predisposition, may overwhelm any potential signals from low-level VC exposure^[^21,22^]^.

The temporal relationship between exposure and disease development warrants further consideration. Our cross-sectional design assessed exposure and outcomes concurrently, thereby potentially missing important latency periods between exposure and comorbidity development. Chronic liver diseases typically evolve over years to decades, and single-year exposure assessments may not capture the cumulative exposure burden relevant to disease pathogenesis^[^23^]^.

With 524 total participants distributed across 12 states and a relatively narrow range of ambient VC concentrations, the study has limited statistical power to detect effect sizes smaller than those observed in occupational cohorts. The absence of statistically significant associations in this study should therefore be interpreted as an absence of detectable evidence of an association at current ambient exposure levels and with the available sample size, rather than as definitive evidence that no association exists.

### Future research directions and study expansion

The analysis focused on the 12 states with available All of Us data. Future studies should consider incorporating data from all available states to enhance the statistical power and geographic representativeness of count-based Poisson regression analyses. This expansion may reveal regional incidence patterns or exposure-response relationships not apparent in the current subset.

Future studies should also consider individual-level exposure assessment methods, including personal exposure monitoring or residential proximity analyses, to better characterize the exposure-response relationships. A longitudinal study design would enable the assessment of temporal relationships and cumulative exposure effects.

Investigations of specific vulnerable subpopulations, such as individuals with genetic polymorphisms affecting VC metabolism, may reveal susceptibility patterns that are not apparent in population-level analyses. Additionally, the examination of other environmental exposures and their potential interactions with VC could provide a more comprehensive understanding of the environmental determinants of liver diseases.

### Clinical and public health implications

Our findings suggest that clinicians caring for HCC patients should focus on established risk factor modifications, such as antiviral therapy for viral hepatitis, alcohol cessation, weight management, and treatment of metabolic syndrome, rather than assessing low-level environmental VC exposure. The high prevalence of hepatic fibrosis and diabetes in this population underscores the importance of comprehensive metabolic evaluations and multidisciplinary care approaches^[^24^]^.

For patients with a known or suspected history of VC exposure, particularly through occupational routes, targeted surveillance for liver-related complications remains appropriate based on established occupational health guidelines^[^25^]^. However, our results do not support routine environmental exposure assessments for patients with HCC in the absence of specific exposure concerns.

From a population health perspective, our findings provide reassurance regarding current environmental VC exposure levels and liver-related health outcomes. However, continued monitoring of environmental exposures and health outcomes remains important, particularly as industrial activities and climate change may influence future exposure patterns^[^26^]^.

The burden of liver-related comorbidities observed in our HCC cohort highlights the continued importance of primary prevention strategies targeting established risk factors such as viral hepatitis, alcohol consumption, and metabolic syndrome. Public health interventions addressing these modifiable risk factors may have a greater population-level impact than current environmental exposure reduction efforts^[^27^]^.

### Role of artificial intelligence and large language models in environmental health research

The growing integration of large language model (LLM) artificial intelligence tools, particularly ChatGPT, into medical and epidemiological research warrants discussion in the context of this study. A recent global-scale bibliometric analysis of ChatGPT in medicine identified substantial growth in LLM-related publications, with a notable concentration in fields intersecting data-intensive methodologies and clinical translation^[^28^]^. This bibliometric landscape is particularly relevant to environmental epidemiology studies such as ours, where large national databases (e.g., the NIH All of Us Research Program) and multi-level statistical analyses are increasingly employed. The authors highlighted current concerns, including reproducibility, data hallucination, and the need for validation of AI-assisted outputs^[^28^]^. Furthermore, as LLMs become embedded in clinical decision support and population health surveillance, environmental health researchers should evaluate their potential roles in automating covariate selection, natural language processing of electronic health records, and geographic exposure linkage. Future directions could incorporate LLM-driven text mining of clinical notes for more granular comorbidity phenotyping beyond SNOMED-CT codes, while maintaining the cautious, human-oversight-centered approach noted in the Artificial Intelligence Declaration of this manuscript^[^28^]^.

## Conclusion

This cross-sectional analysis of 524 HCC patients showed a high prevalence of hepatic fibrosis (82.8%) and diabetes (55.0%). Poisson regression analyses, offset by the state-level All of Us participant population size, demonstrated a significant association between hepatic fibrosis status and case counts (IRR = 4.96, 95% CI: 3.88–6.42, *P* < 0.001), while diabetes status showed no significant association (IRR = 1.17, 95% CI: 0.97–1.42, *P* = 0.094). Individual-level multinomial logistic regression, controlling for potential confounders, showed no meaningful associations between VC exposure and comorbidity patterns.

Although liver-related comorbidities represent a substantial burden in HCC populations, the significant Poisson regression finding for hepatic fibrosis reflects the strong underlying epidemiological association between fibrosis and HCC rather than a VC-driven effect. Current environmental VC exposure levels do not appear to be significant determinants of comorbidity patterns.

These results support the continued focus on risk factor modification and comprehensive comorbidity management in HCC patient care. Future research employing individual-level exposure assessments, longitudinal study designs, and expanding to all available states using Poisson regression frameworks may provide additional insights into the potential relationships between environmental VC exposure and liver-related health outcomes. However, the current evidence does not support routine environmental exposure assessments for patients with HCC in the absence of specific exposure concerns.

## Data Availability

The data that support the findings of this study are available from the NIH All of Us Research Program. Restrictions apply to the availability of these data, which were used under license for this study. Data are available from the All of Us Researcher Workbench for researchers who meet the criteria for access.

## Presentation

Preliminary findings from this study were presented at the 21st Annual Academic Surgical Congress.
